# Molecular Identification of Piroplasmids in Ticks from Infested Small Ruminants in Konya Province, Turkey

**DOI:** 10.3390/pathogens12091123

**Published:** 2023-09-01

**Authors:** Zhuowei Ma, Onur Ceylan, Eloiza May Galon, Uday Kumar Mohanta, Shengwei Ji, Hang Li, Thanh Thom Do, Rika Umemiya-Shirafuji, Shimaa Abd El-Salam El-Sayed, Iqra Zafar, Mingming Liu, Ferda Sevinc, Xuenan Xuan

**Affiliations:** 1 National Research Center for Protozoan Diseases, Obihiro University of Agriculture and Veterinary Medicine, Obihiro 080-8555, Japan; mazhuowei1994@gmail.com (Z.M.); uday_vet01@yahoo.com (U.K.M.); jishengwei0903@hotmail.com (S.J.); lihang-2020@hotmail.com (H.L.); thanhthomdo@gmail.com (T.T.D.); umemiya@obihiro.ac.jp (R.U.-S.); shimaa_a@mans.edu.eg (S.A.E.-S.E.-S.); eekrawahla@hotmail.com (I.Z.); gen@obihiro.ac.jp (X.X.); 2 Department of Parasitology, Faculty of Veterinary Medicine, Selcuk University, 42250 Konya, Turkey; onurceylan@selcuk.edu.tr; 3 College of Veterinary Medicine and Biomedical Sciences, Cavite State University, Indang 4122, Philippines; eloiza.galon@cvsu.edu.ph; 4 Department of Microbiology and Parasitology, Sher-e-Bangla Agricultural University, Sher-e-Bangla Nagar, Dhaka 1207, Bangladesh; 5 Department of Biochemistry and Chemistry of Nutrition, Faculty of Veterinary Medicine, Mansoura University, Mansoura 35516, Egypt; 6 School of Basic Medicine, Hubei University of Arts and Science, Xiangyang 441053, China

**Keywords:** piroplasmids, tick species, molecular identification, Konya Province, Turkey

## Abstract

Ticks play a pivotal role in propagating a diverse spectrum of infectious agents that detrimentally affect the health of both humans and animals. In the present study, a molecular survey was executed of piroplasmids in ticks collected from small ruminants in four districts within Konya province, Turkey. Microscopic examination identified 1281 adult ticks, which were categorized into 357 pools based on their species, sexes, host animals, and collection site before DNA extraction. The infection rates were calculated by using a maximum likelihood estimate (MLE) with 95% confidence intervals (CI). *Hyalomma detritum*, *H*. *excavatum*, *Rhipicephalus bursa*, *R*. *sanguineus*, and *R*. *turanicus* were identified in this study. Among the five tick species identified here, *R*. *turanicus* exhibited the highest infestation rate in both goats and sheep. The presence of *Babesia ovis* and *Theileria ovis* based on 18S rRNA was confirmed using molecular assay. The overall MLE of infection rates for *B*. *ovis* and *T*. *ovis* was 2.49% (CI 1.72–3.46) and 1.46% (CI 0.87–2.23), respectively. The MLE of *B*. *ovis* and *T*. *ovis* infection rates in *R*. *bursa* was 10.80% (CI 7.43–14.90) and 0.33% (CI 0.02–1.42), respectively, while that in *R*. *turanicus* was 0.12% (CI 0.01–0.51) and 2.08% (CI 1.25–3.22). This study further confirms that *R*. *turanicus* and *R*. *sanguineus* can act as vectors for *B*. *ovis*, thus advancing our comprehension of tick-borne piroplasmids epidemiology and providing valuable insights for the development of effective control strategies for ticks and tick-borne diseases in Turkey.

## 1. Introduction

Piroplasms, *Babesia* spp., and *Theileria* spp., which are conveyed by ticks, serve as the etiologic agents of piroplasmosis that significantly impact the economy of the livestock industry, resulting in production losses, treatment and prevention costs, and increased morbidity and mortality [1,2,3]. Babesiosis and theileriosis are found in different hosts and are generally prevalent in tropical and subtropical zones [4]. *T*. *parva* and *T*. *annulata* cause bovine clinical theileriosis, while *T*. *buffeli*, *T*. *orientalis*, and *T*. *sergenti* are associated with benign theileriosis [5]. *B*. *bovis*, *B*. *bigemina*, *B*. *divergens*, *B*. *major*, *B*. *occultans*, and *B*. *ovata* are confirmed to infect cattle; however, only *B*. *bovis*, *B*. *bigemina*, and *B*. *divergens* are known to cause clinical babesiosis [6]. Moreover, a recent study provided evidence that *B*. *naoakii* (*Babesia* sp. Mymensingh) can cause acute clinical babesiosis in cattle [7]. In small ruminants, *T*. *lestoquardi*, *T*. *luwenshuni*, and *T*. *uilenbergi* are considered the pathogenic *Theileria* spp. causing malignant theileriosis [8,9]. On the other hand, *B*. *ovis*, *B*. *motasi*, *B*. *crassa*, and *Babesia* sp. Xinjiang are the causative agents that can induce severe babesiosis [10,11].

Ticks are essential hematophagous arachnid external parasites affiliated with the Ixodida suborder, deemed inferior only to mosquitoes in relevance as carriers of pathogenic entities significantly impacting animal and human health [12,13,14,15]. Ticks exhibit a broad spectrum of hosts, encompassing mammals, birds, and reptiles; during their blood meal, ticks can transmit a multitude of pathogens to their hosts [16,17]. According to the recent classification, the tick species are categorized into three families: Ixodidae, which included 702 species of hard ticks; Argasidae, including 193 species of soft ticks; and Nuttalliellidae, represented solely by *Nuttalliella namaqua* [18]. The prevalence of ticks fluctuates with the season [19,20]. While most tick species persist in low numbers throughout the year, their abundance spikes briefly under favorable climatic conditions. In 1984, the United Nations Food and Agricultural Organization (FAO) estimated that Ixodidae tick infestations resulted in a staggering global cost of $US 7.0 billion annually due to skin damage and tick-borne diseases [21].

Owing to the unique climatic conditions of Turkey, diverse vegetation, and fertile soil, the country possesses a substantial livestock resource, with small ruminants representing the primary asset and their total population exceeding 56 million in animal numbers [22,23]. These conditions within the country also provide a conducive environment for ticks and facilitate the maintenance of parasite-host-vector relationships [4,24]. To date, in Turkey, 51 tick species have been identified (43 from Ixodidae and 8 from Argasidae families) [25]. The most frequently encountered tick genera in this country include *Hyalomma*, *Ixodes*, *Haemaphysalis*, *Dermacentor*, and *Rhipicephalus* [26,27,28,29,30,31,32]. Several studies performed the epidemiological analysis of *Babesia* spp. and *Theileria* spp. in small ruminants in different regions of Turkey [8,33,34,35,36]. A comprehensive comprehension of tick-borne piroplasmid epidemiology is vital for disease control. However, data on tick vectors of piroplasmids infecting small ruminants in Turkey are still limited, and some provinces have not yet reported on occurrences of tick-borne piroplasmids. Therefore, this study aimed to conduct the molecular identification of *Theileria* and *Babesia* species in ixodid ticks collected from sheep and goats to enhance the understanding of their distribution in Turkey for their effective control.

## 2. Materials and Methods

### 2.1. Ethical Statement

The sheep and goat owners were duly informed about the research goals, and their consent was obtained before the tick samples collection. All procedures related to tick sampling and subsequent processing were as per the ethical guidelines established by Obihiro University of Agriculture and Veterinary Medicine. (Animal experiment approval number: 19–74; DNA experiment approval number: 1706-4, 1705-4, 1704-4).

### 2.2. Study Area

**Figure 1:**
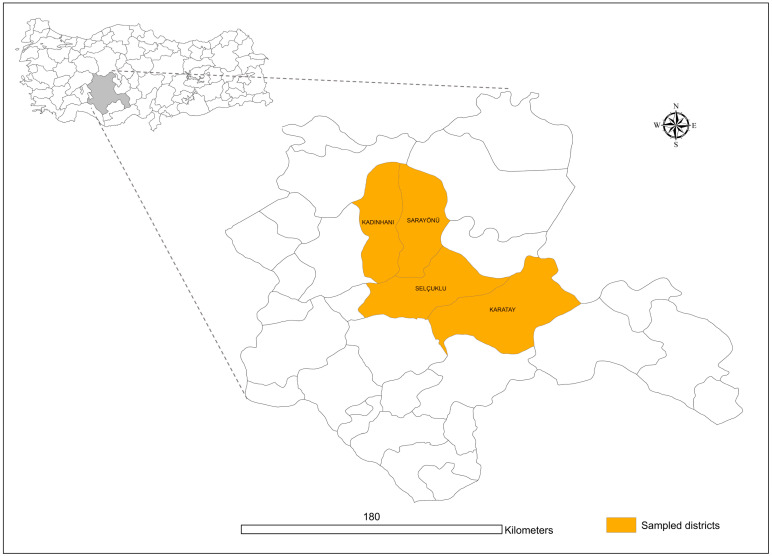
Map showing the four districts (Kadınhanı, Karatay, Sarayönü, Selçuklu) of Konya Province, Turkey, where tick samples were collected.

Konya, located between 36°41′ and 39°16′ north latitude and 31°14′ and 34°26′ east longitude, is the province with the largest land area in the southern part of the Central Anatolia region of Turkey. Its surface area is 38,873 km^2^ (excluding lakes). The average elevation is 1.016 m. Konya’s provincial territory has 60% cultivated and planted areas, 17% forests and heathlands, and 15% meadows and pastures. Konya resembles a big steppe. There is little forest. Konya province has harsh, cold, snowy winters and hot and dry summers. The average annual temperature is 11.5 °C, and the average relative humidity is 60. The average annual precipitation in Konya is 326 mm. Although only a few districts of Konya close to the Mediterranean region have a slightly Mediterranean climate, the districts of Kadınhanı, Karatay, Sarayönü, and Selçuklu (Figure 1), where ticks were collected in the study have the main climatic, vegetational, and geographical characteristics found in the province.

### 2.3. Tick Collection and Identification

In 2013, tick samples were gathered between May and June. 614 and 667 ticks were randomly collected from apparently healthy 76 sheep and 29 goats, respectively. Each tick was carefully removed from the host’s skin with the livestock owner’s permission using a fine-tipped bent tweezer, making sure it was close to the dermis to minimize any harm and discomfort for the species. All tick specimens were then preserved in 70% ethanol. The identification of tick species was conducted employing a binocular microscope (Olympus SZX16, Tokyo, Japan), following the guidelines of standard taxonomic keys [37].

### 2.4. DNA Extraction

Upon microscopic identification, the tick specimens were grouped based on species, sexes, and site of collection, culminating in the formation of 357 pools, with each pool containing 1 to 10 ticks, guided by the sample size. The tick DNA was extracted following the methodology previously described by [38], using a NucleoSpin Tissue Kit (Macherey-Nagel, Düren, Germany), following the manufacturer’s instructions. The extracted DNA was eluted with 50 μL of double-distilled water (Invitrogen, UltrapureTM Distilled Water, DNAse, and RNAse, Free) and then preserved at −30 °C awaiting further utilization.

### 2.5. Molecular Identification of Tick-Borne Piroplasmids

All tick pool specimens underwent initial screening with a universal primer target at the 18S rRNA genes of both *Babesia* and *Theileria* [39], with a thermocycling protocol began with an initial denaturation at 95 °C for 3 min, followed by 35 cycles of 30-s denaturation at 95 °C, 30-s annealing at 55 °C, and 1 min extension at 72 °C. A final extension step was carried out at 72 °C for 7 min. The positive samples obtained from the initial screening were chosen for species-specific identification. The partial regions of several genes were amplified, including the apical membrane antigen-1 (*AMA-1*) of *B*. *naoakii*, the 18S rRNA of *B*. *ovis*, the merozoite surface antigen (*Tlms*) of *T*. *lestoquardi*, the 18S rRNA of *T*. *luwenshuni*, the 18S rRNA of *T*. *ovis*, and the 18S rRNA of *T*. *uilenbergi*. The PCR cycling conditions for species-specific piroplasmids screening mirrored those previously described, with the only variation being the annealing temperatures, which were adopted from the referenced publications and are elaborated upon in Table 1. The 10 µL final reaction mixture was used for all PCR screening that contained 1 µL of 10× AmpliTaq gold 360 buffer (Applied Biosystems, Waltham, MA, USA), 0.8 µL of 25 mM magnesium chloride (Applied Biosystems), 0.8 µL of dNTP mix (Applied Biosystems), 0.2 µM of each primer, 0.05 µL of 5 U/µL AmpliTaq gold 360 DNA polymerase (Applied Biosystems), 1 µL of DNA template, and 5.95 µL of double-distilled water. Positive controls included previously sequence-confirmed DNA templates and double-distilled water was used as the negative control.

### 2.6. Cloning and Sequencing Analysis

Selected partial positive PCR amplicons, based on the presence of piroplasmids detected from various tick species, were chosen for sequencing. To ensure adequate concentrations for sequencing, PCR was conducted in larger volumes. PCR amplicons were purified employing a gel extraction kit. (NucleoSpinTM Gel and PCR Clean Up, Macherey-Nagel, Germany). The concentration of eluted DNA was measured using a NanoDrop 2000 spectrophotometer. All PCR amplicons with an adequate DNA concentration were directly sequenced, and samples of low concentration were first cloned into a pGEM vector as per the commercial protocol of pGEM^®^-T Easy Vector System (Promega, Fitchburg, WI, USA). Recombinant clones resulting from this transformation were selected for sequencing. The plasmid was extracted from this culture using the Nucleospin^®^ Plasmid QuickPure Kit (Macherey-Nagel-German), and the sequencing of samples was performed with the Big Dye Terminator v3.1 Cycle Sequencing Kit (Applied Biosystems) on an ABI PRISM 3130xl Genetic Analyzer (Applied Biosystems).

### 2.7. Phylogenetic and Statistical Analysis

Nucleotide sequences garnered in this investigation were aligned with those previously submitted to GenBank via the BLASTn algorithm, facilitating the determination of identities and similarities. Phylogenetic trees for *B*. *ovis* (18S rRNA) and *Babesia*/*Theileria* (18S rRNA) were prepared using the maximum likelihood method facilitated using MEGA version X software. Bootstrap analysis with 1000 replications was employed to ascertain the confidence of the nodes and branches within the trees. Finally, sequences derived from the current study were submitted to the GenBank of the National Center for Biotechnology Information via BankIt. Statistical analysis was performed in R software, version 4.3.1 (The R Foundation for Statistical Computing). The maximum-likelihood estimate (MLE) of pooled prevalence was calculated using the “PoolTestR” package [40].

### 2.8. Accession Numbers

Sequences derived from this research have been submitted to GenBank and can be accessed using the following accession numbers: for *B*. *ovis*: OR394130, OR395165, OR395166, OR395167, OR395168, OR395169, and OR395170; for *T*. *ovis*: OR395235, OR395236, OR395237, OR395238, OR395239, OR395240, OR395241, OR395242, OR395243, OR395244, OR395245, OR395246, OR395247, OR395248, OR395249, OR395250, OR395251, and OR395252.

**Table 1:** PCR primers were used for the piroplasmids identification in this study.

Target Piroplasmid	Target Gene	Primer Sequence (5′ to 3′)	Size (bp)	Annealing Temperature (°C)	References
*Babesia*/*Theileria* spp.	18S rRNA	GTCTTGTAATTGGAATGATGG TAGTTTATGGTTAGGACTACG	~500	55	[39]
*B*. *naoakii*	*AMA-1*	TGGCGCCGACTTCCTGGAGCCCATCTCCAA AGCTGGGGCCCTCCTTCGATGAACCGTCGG	371	64	[41]
*B*. *ovis*	18S rRNA	TGGGCAGGACCTTGGTTCTTCT CCGCGTAGCGCCGGCTAAATA	549	62	[10]
*T*. *lestoquardi*	Merozoite surface antigen (*Tlms*)	GTGCCGCAAGTGAGTCA GGACTGATGAGAAGACGATGAG	730	54	[42]
*T*. *luwenshuni*	18S rRNA	GGTAGGGTATTGGCCTACTGA TCATCCGGATAATACAAG	389	57	[43]
*T*. *ovis*	18S rRNA	TCGAGACCTTCGGGT TCCGGACATTGTAAAACAAA	520	53	[44]
*T*. *uilenbergi*	18S rRNA	GGTAGGGTATTGGCCTACCGG ACACTCGGAAAATGCAAGCA	388	55	[43]

## 3. Results

### 3.1. Tick Species Identification and Infestation Rate

In this study, morphological identification showed that the 1281 ixodid adult ticks gathered from 76 sheep and 29 goats belonged to two genera, *Hyalomma* and *Rhipicephalus*; among these, five species, *H*. *detritum* (n = 7), *H*. *excavatum* (n = 94), *R*. *bursa* (n = 308), *R*. *sanguineus* (n = 3), and *R*. *turanicus* (n = 869) were from the four districts of Konya province. The heaviest tick infestation in sheep and goats was observed in the Selçuklu district, followed by the Kadınhanı district. 

**Figure 2:**
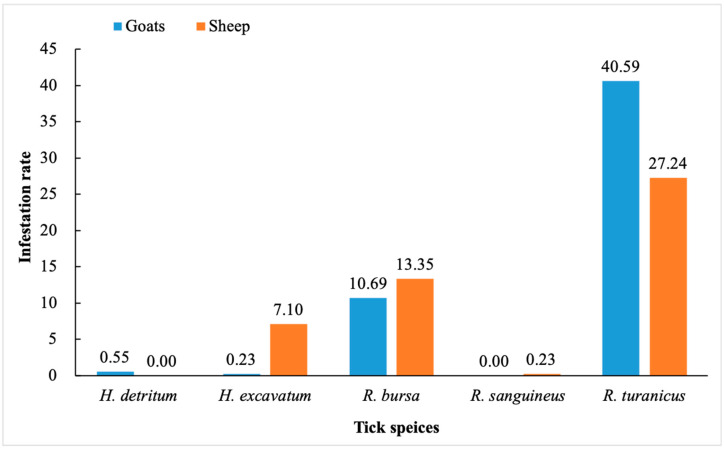
Total infestation rate of *H*. *detritum*, *H*. *excavatum*, *R*. *bursa*, *R*. *sanguineus*, and *R*. *turanicus* in small ruminants in this study.

The infestation rates of *H*. *detritum*, *H*. *excavatum*, *R*. *bursa*, *R*. *sanguineus*, and *R*. *turanicus* were 0.55, 0.23, 10.69, 0, and 40.59% in goats, and 0, 7.10, 13.35, 0.23, and 27.24% in sheep, respectively (Figure 2). 

### 3.2. Detection of Piroplasms in Ticks

Among the 357 tick pools analyzed in this study, 49 pools had piroplasms. *B. ovis* and *T. ovis* were identified in 31/357 and 18/357 pools, respectively, and no other piroplasma species were detected. The MLE for *B. ovis* was 2.49% (CI 1.72–3.46), while for *T*. *ovis*, it was 1.46% (CI 0.87–2.23).

Three tick species, *R*. *bursa*, *R. sanguineus*, and *R. turanicus* were detected that carried *Babesia* or *Theileria* in which *B. ovis* was present in all three species; in contrast, *T. ovis* was identified only in *R. bursa* and *R. turanicus*. The MLE of *B. ovis* in *R. bursa* (29/118 pools) and *R. turanicus* (1/169 pools) were 10.80% (CI 7.43–14.90) and 0.12% (CI 0.01–0.51), respectively. Due to significant variation in the number of pools for *R. sanguineus* compared to that of the other two species, the MLE for *B. ovis* in *R. sanguineus* pools was not estimated. Furthermore, the estimated MLE for *T. ovis* in *R. bursa* (1/118 pools) and *R. turanicus* (17/169 pools) was 0.33% (CI 0.02–1.42) and 2.08% (CI 1.25–3.22), respectively.

The presence of piroplasma was noted across all four examined districts. *B. ovis* was absent in the Kadınhanı district but was identified in the other three districts. *T. ovis* was found in the Kadınhanı and Selçuklu districts. A detailed distribution of piroplasma detection rates, based on district and tick species, is presented in Table 2.

### 3.3. Phylogenetic Analyses of B. ovis and T. ovis

**Figure 3:**
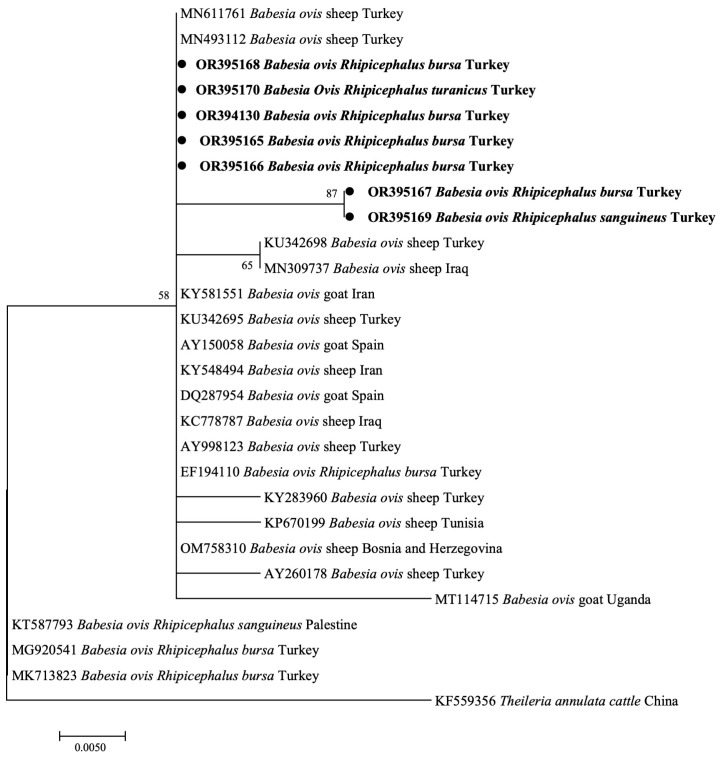
Phylogenetic analysis of *B*. *ovis* based on the 18S rRNA. Sequences identified in the current investigation are highlighted in bold. Node numbers indicate the percentage frequency of clades across 1000 bootstrap replicates of the taxa.

In this study, phylogenetic trees using the *B*. *ovis* 18S rRNA (BoSSUrRNA) gene sequence were constructed. The BoSSUrRNA sequences of *B*. *ovis* from *R*. *bursa*, *R*. *sanguineus*, and *R*. *turanicus*, identified in this study, were phylogenetically clustered within the same clade as sheep and tick isolates from Turkey, and sheep and goat isolates from various other countries, including Bosnia and Herzegovina, Iran, Iraq, Spain, Tunisia, and Uganda. However, three sequences (KT587793, MG920541, MK713823) exhibited divergent clustering from *R*. *sanguineus* and *R*. *bursa* in Palestine and Turkey, which formed a separate clade (Figure 3). 

**Figure 4:**
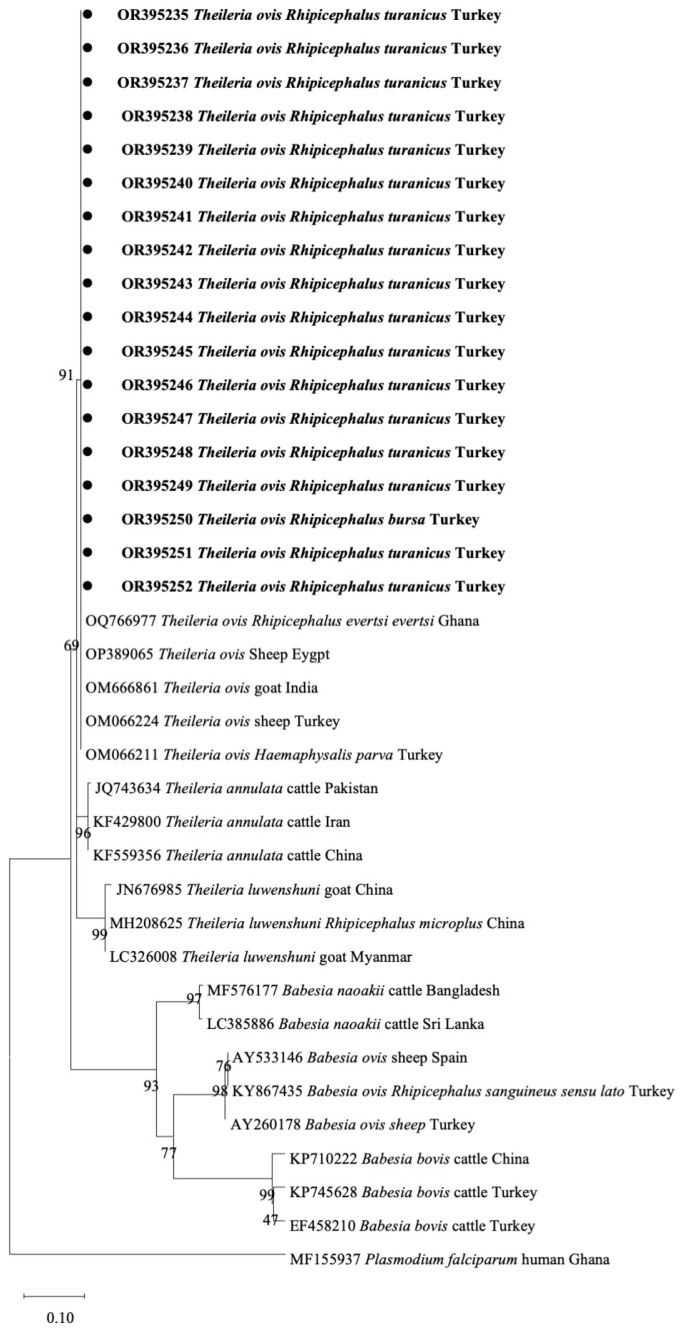
Phylogenetic analysis of *Babesia* and *Theileira* species based on the 18S rRNA. Sequences identified in the current investigation are highlighted in bold. Node numbers indicate the percentage frequency of clades across 1000 bootstrap replicates of the taxa.

Eighteen positive samples for the *Babesia*/*Theileria* 18S rRNA gene using a universal primer; however, these were negative when species-specific primers were used and included in a phylogenetic tree prepared using the universal primer for the 18S rRNA gene. These eighteen samples formed a distinct clade and clustered with *T*. *ovis* sequences from Ghana (OQ7669770), Egypt (OP389065), India (OM666861), and Turkey (OM066224, OM066211). This clade was distinct from *T*. *luwenshuni*, *T*. *annulata*, *B*. *naoakii*, *B*. *ovis*, and *B*. *bovis* sequences from samples of other countries (Figure 4).

## 4. Discussion

Ticks are pivotal in veterinary and human health, serving as carriers for a variety of infectious agents, which include bacteria, helminths, protozoa, and viruses. In recent years, Turkey has documented an uptick in the incidence of tick-borne diseases, a phenomenon also observed in numerous regions globally [45]. This increase is significantly influenced by climate change, which alters tick-friendly ecological niches and might lengthen tick’s active seasons. A further level of intricacy is added by avian migration patterns. Birds can spread ticks across large geographic distances, possibly introducing or increasing the incidence of ticks-associated pathogens. [46]. Notably, *Babesia* spp. and *Theileria* spp., tick-borne piroplasmids, rank the most hemoparasites impacting the economy of small ruminant industries, with a worldwide distribution [1]. To mitigate tick-borne piroplasmids transmission, it is imperative to understand the intricate associations between ticks and piroplasms, given their significance to public health. In the present study, we conducted a molecular examination of piroplasmids in ticks collected from small ruminants in four districts (Kadınhanı, Karatay, Sarayönü, Selçuklu) in the Konya province of Turkey. Microscopic examination revealed two genera, *Hyalomma* and *Rhipicephalus*, including five species: *H*. *detritum*, *H*. *excavatum*, *R*. *bursa*, *R*. *sanguineus*, and *R*. *turanicus*. Molecular assays further identified two piroplasms, *B*. *ovis*, and *T*. *ovis*. 

Climatic factors like rainfall, vegetation, altitude, and temperature, along with host availability, influence the distribution trends of ixodid ticks and the spread of tick-borne diseases. [47]. Tick infestation in sheep and goats in Turkey is common due to geographic conditions and management systems [4]. In the present study, both goats and sheep exhibited a pronounced infestation rate of *R*. *turanicus*, with 40.59% in goats and 27.24% in sheep, followed by *R*. *bursa* (goats: 10.69%; sheep: 13.35%). A previous study identified *R*. *turanicus*, *Ha*. *parva*, and *R*. *bursa* as the predominant species of sheep and goats in the Black Sea Region of Turkey [32]. Ceylan et al. 2021 [48] reported *R*. *turanicus* as the most prevalent tick species among sheep in Turkey, consistent with our finding. Interestingly, *H*. *excavatum* demonstrated a relatively higher infestation rate (7.10%) in our study compared to those conducted earlier [32,48] and *Ha*. *parva* was not detected, possibly attributable to geographical and seasonal variations. Of note, despite the comparable climatic conditions across the four selected districts, the presence of *R*. *turanicus* was solely identified in the Kadınhanı district. To comprehensively understand the influence of geographical disparities across the remaining districts, in-depth investigations are warranted. Such studies would contribute valuable insights into vector distribution patterns and potential ramifications for disease transmission dynamics.

The most important *Babesia* species associated with babesiosis in small ruminants are considered *B*. *ovis*, *B*. *crassa*, *B*. *motasi*, and *Babesia* sp. Xinjiang [11,49,50]. Among these, *B*. *ovis* is particularly pathogenic in sheep and goats [51], and ovine babesiosis caused by this piroplasma exhibits a state of nationwide endemic instability in Turkey [52]. Although molecular detection of *B*. *ovis* in sheep and goats has been reported in various provinces of Turkey [8,35,53,54], no earlier study had identified tick vectors of this parasite in the Konya province. In this study, we discovered that *R*. *bursa*, *R*. *sanguineus*, and *R*. *turanicus* can serve as vectors for *B*. *ovis* in this region. This parasite is documented to be carried by *R*. *bursa*, *R*. *sanguineus*, *R*. *turanicus*, *Ha*. *parva*, *H*. *marginatum*, and *H*. *excavatum* [31,55,56,57]. Our findings indicate that *B*. *ovis* was detected in 29 out of 118 *R*. *bursa* pools (10.80%, CI 7.43–14.90), 1 out of 169 *R*. *turanicus* pools (0.12%, CI 0.01–0.51), and 1 out of 3 *R*. *sanguineus* pools. This observation particularly of *B*. *ovis* in *R*. *bursa*, is consistent with that of prior studies [31,55]. There is one report each of the detection of *B*. *ovis* in *R*. *turanicus* and *R*. *sanguineus* in Turkey [55,57]. The present study substantiates these findings, suggesting that *R*. *turanicus* and *R*. *sanguineus* may act as vectors for *B*. *ovis* in this country. Moreover, phylogenetic analyses using 18S rRNA gene sequences formed two diverged clades of *B*. *ovis*. Further investigation into the genetic diversity of *B*. *ovis* is warranted. Molecular diagnostic techniques employed in the study of *Babesia* species have facilitated the identification of previously unrecognized species, including *B*. *naoakii*, implicated in cases of acute babesiosis [7]. Although its incidence in a few nations has been verified, no reports have indicated that it exists in Turkey. One of our goals in doing this experiment was to determine whether this piroplasmid might exist in Turkey. All samples, though, came out negative for this parasite. A recent study provided molecular evidence that *B*. *naoakii* was detected in *Ha*. *bispinosa* [58]. At the same time, there is no current evidence to suggest the presence of *Ha*. *bispinosa* in Turkey, the transmission of pathogens might be mediated by another tick vector, especially given the absence of the primary vector in the region [59]. Consequently, future studies should be maintaining a monitor of this parasite in both mammalian and potential vector hosts in the country.

Due to their varied pathogenic effects on small ruminants, *Theileria* species have long been a focus of research. *T*. *lestoquardi*, *T*. *luwenshuni*, and *T*. *uilenbergi* are known to cause severe theileriosis, which can harm the health and production of these animals. In contrast, *T*. *ovis* is generally considered a non-pathogenic species of *Theileria* [8]. However, it is noteworthy that *T. ovis* can switch from a benign to a pathogenic state under certain predisposing factors, primarily stressful situations or physiological states, leading to severe theileriosis. [60]. Therefore, it should not be ignored in the epidemiology. Past research in Turkey has reported the presence of *T*. *ovis*, *T*. *luwenshuni*, *T*. *uilenbergi*, and certain *Theileria* isolates, such as *Theileria* sp. MK, *Theileria* sp. OT1, and *Theileria* sp. OT3 in small ruminants [8,33,61]. Consequently, it is expected that the vectors of these piroplasmids are expected to exist within the country. However, in our study, only *T*. *ovis* was identified in the *Rhipicephalus* ticks, possibly because the primary transmission vector was not collected in this study [62]. The prevalence of *T*. *ovis* in *R*. *bursa* and *R*. *turanicus* pools were 0.33% (1/118, CI 0.02–1.42) and 2.08% (17/169, CI 1.25–3.22), respectively. In a prior study from the same province as our tick sampling site, *T*. *ovis* was detected in sheep with an infection rate of 21.05% [36], which is relatively higher than our current findings. As documented in a previous study, *Rhipicephalus* ticks are the vectors for *T*. *ovis* transmission in Turkey [53], consistent with the results of this study. The vector for carrying piroplasmids may vary due to geographical differences. Therefore, future studies should explore the relationship between tick species and *Theileria* spp., which were not detected in this study, should be explored in future studies to provide more comprehensive information for controlling theileriosis.

*B*. *ovis* has been detected in all developmental stages of *R*. *bursa*, demonstrating both transovarial and transstadial transmission capabilities, as previously documented [63]. In contrast, *T*. *ovis* is acquired by the immature stages of *R*. *bursa* and transmitted by the subsequent adults [64]. Hence, the vital role of tick larvae and nymphs in piroplasm transmission is well-established. However, in our study, only adult ticks were collected. To provide a comprehensive understanding of piroplasmid prevalence, future investigations should prioritize the assessment of piroplasmids across various life stages of ticks. It is worth noting that no existing reports detail the development and transmission of *B*. *ovis* and *T*. *ovis* in *R*. *turanicus* and *R*. *sanguineus*. As a result, further research is warranted to delve into the intricate dynamics of *B*. *ovis* and *T*. *ovis* development and transmission in these tick species.

## 5. Conclusions

In conclusion, we investigated the distribution and prevalence of tick-borne piroplasmids in ticks infesting sheep and goats within the Konya province of Turkey, a region where no previous reports identified tick vectors for such piroplasmids. The presence of *B*. *ovis* and *T*. *ovis* was confirmed in tick vectors in this province. Furthermore, this study corroborates that *R*. *turanicus* and *R*. *sanguineus* can serve as potential vectors for *B*. *ovis* transmission in this region. This research augments our comprehension of the epidemiology of piroplasmids in tick vectors. This research augments our comprehension of the epidemiology of piroplasmids in tick vectors and is expected to contribute valuable insights toward the development of effective strategies for the control of ticks and tick-borne diseases.

## Figures and Tables

**Table 2:** The collected tick distribution and infection rates of *B. ovis* and *T. ovis* within the four districts in Konya province.

District	Tick Species (No. of Tick/No. of Tick Pool)	*B*. *ovis* (No. of Tick/No. of Tick Pool)	*T*. *ovis* (No. of Tick/No. of Tick Pool)
Kadınhanı	*R*. *turanicus* (547/89)	-	12/89 (2.33% ^a^) CI ^b^ (1.26–3.88)
Karatay	*R*. *bursa* (3/1)	1/1	-
	*R*. *sanguineus* (1/1)	1/1	-
Sarayönü	*R*. *bursa* (127/40)	7/40 (6.24%) CI (2.72–11.80)	-
	*R*. *sanguineus* (39/12)	-	-
Selçuklu	*H*. *detritum* (7/7)	-	-
	*H*. *excavatum* (94/60)	-	-
	*R*. *bursa* (178/77)	21/77 (13.50%) CI (8.73–19.60)	1/77 (0.56%) CI (0.03–2.45)
	*R*. *sanguineus* (2/2)	-	-
	*R*. *turanicus* (283/68)	1/68 (0.36%) CI (0.02–1.59)	5/68 (1.90%) CI (0.69–4.04)
Total	*R*. *bursa* (308/118)	29/118 (10.80%) CI (7.43–14.90)	1/118 (0.33%) CI (0.02–1.42)
	*R*. *turanicus* (869/169)	1/169 (0.12%) CI (0.01–0.51)	17/169 (2.08%) CI (1.25–3.22)

^a^: The rate of MLE; ^b^: 95% confidence interval.

## Data Availability

The datasets generated during and/or analyzed during the current study are available from the corresponding author upon reasonable request.

## References

[1] Uilenberg G. (1995). International collaborative research: Significance of tick-borne hemoparasitic diseases to world animal health. Vet. Parasitol..

[2] Minjauw B., McLeod A. (2003). Tick-borne diseases and poverty: The impact of ticks and tick-borne diseases on the livelihoods of small-scale and marginal livestock owners in India and Eastern and Southern Africa. Tick-Borne Diseases and Poverty: The Impact of Ticks and Tick-Borne Diseases on the Livelihoods of Small-Scale and Marginal Livestock Owners in India and Eastern and Southern Africa.

[3] McLeod R., Kristjanson P. (1999). Economic Impact of Ticks and Tick-Borne Diseases to Livestock in Africa, Asia and Australia.

[4] Ceylan O., Xuan X., Sevinc F. (2021). Primary tick-borne protozoan and rickettsial infections of animals in Turkey. Pathogens.

[5] Minami T., Fujinaga T., Furuya K., Ishihara T. (1980). Clinico-hematologic and serological comparison of Japanese and Russian strains of *Theileria sergenti*. Natl. Inst. Anim. Health Q..

[6] Bock R., Jackson L., de Vos A., Jorgensen W. (2004). Babesiosis of cattle. Parasitology.

[7] Sivakumar T., Tuvshintulga B., Otgonsuren D., Batmagnai E., Ahedor B., Kothalawala H., Vimalakumar S.C., Silva S.S.P., Yamagishi J., Yokoyama N. (2022). Phylogenetic analyses of the mitochondrial, plastid, and nuclear genes of *Babesia* sp. Mymensingh and its naming as *Babesia naoakii* n. sp. Parasit. Vectors.

[8] Bilgic H.B., Bakırcı S., Kose O., Unlu A.H., Hacılarlıoglu S., Eren H., Weir W., Karagenc T. (2017). Prevalence of tick-borne haemoparasites in small ruminants in Turkey and diagnostic sensitivity of single-PCR and RLB. Parasit. Vectors.

[9] Morel P.C., Uilenberg G. (1981). The nomenclature of some *Theileria* species (Sporozoa, Babesioidea) of domestic ruminants (Author’s Transl). Rev. Elev. Med. Vet. Pays. Trop..

[10] Aktaş M., Altay K., Dumanli N. (2005). Development of a polymerase chain reaction method for diagnosis of *Babesia ovis* infection in sheep and goats. Vet. Parasitol..

[11] Liu A.H., Yin H., Guan G.Q., Schnittger L., Liu Z.J., Ma M.L., Dang Z.S., Liu J.L., Ren Q.Y., Bai Q. (2007). At least two genetically distinct large *Babesia* species infective to sheep and goats in China. Vet. Parasitol..

[12] Köseoğlu A.E., Can H., Güvendi M., Erkunt Alak S., Kandemir Ç., Taşkın T., Demir S., Akgül G., Değirmenci Döşkaya A., Karakavuk M. (2021). Molecular investigation of bacterial and protozoal pathogens in ticks collected from different hosts in Turkey. Parasit. Vectors.

[13] De la Fuente J., Estrada-Pena A., Venzal J.M., Kocan K.M., Sonenshine D.E. (2008). Overview: Ticks as vectors of pathogens that cause disease in humans and animals. Front. Biosci..

[14] Nava S., Guglielmone A.A., Mangold A.J. (2009). An overview of systematics and evolution of ticks. Front. Biosci..

[15] Reuben Kaufman W. (2010). Ticks: Physiological aspects with implications for pathogen transmission. Ticks Tick Borne Dis..

[16] Guo H., Adjou Moumouni P.F., Thekisoe O., Gao Y., Liu M., Li J., Galon E.M., Efstratiou A., Wang G., Jirapattharasate C. (2019). Genetic characterization of tick-borne pathogens in ticks infesting cattle and sheep from three South African provinces. Ticks Tick Borne Dis..

[17] Teng Z., Shi Y., Zhao N., Zhang X., Jin X., He J., Xu B., Qin T. (2023). Molecular detection of tick-borne bacterial and protozoan pathogens in *Haemaphysalis longicornis* (Acari: Ixodidae) ticks from free-ranging domestic sheep in Hebei Province, China. Pathogens.

[18] Guglielmone A.A., Robbins R.G., Apanaskevich D.A., Petney T.N., Estrada-peña A., Horak I.G., Shao R., Barker S.C. (2010). The Argasidae, Ixodidae and Nuttalliellidae (Acari: Ixodida) of the world: A list of valid species names. Zootaxa.

[19] Randolph S.E., Green R.M., Hoodless A.N., Peacey M.F. (2002). An empirical quantitative framework for the seasonal population dynamics of the tick *Ixodes ricinus*. Int. J. Parasitol..

[20] Guglielmone A.A. (1994). The seasonal occurrence of *Amblyomma triguttatum triguttatum* Koch (Acari, Ixodidae). Acarologia.

[21] Abunna F., Kasasa D., Shelima B., Megersa B., Regassa A., Amenu K. (2009). Survey of tick infestation in small ruminants of Miesso district, West Harergie, Oromia Region, Ethiopia. Trop Anim. Health Prod..

[22] Sevinc F., Xuan X. (2015). Major tick-borne parasitic diseases of animals: A frame of references in Turkey. Eurasian J. Vet. Sci..

[23] Turkish Statistical Institute. http://www.turkstat.gov.tr/.

[24] Bursali A., Keskin A., Tekin S. (2012). A review of the ticks (Acari: Ixodida) of Turkey: Species diversity, hosts and geographical distribution. Exp. Appl. Acarol..

[25] Ji S., Ceylan O., Ma Z., Galon E.M., Zafar I., Li H., Hasegawa Y., Sevinc M., Masatani T., Iguchi A. (2022). Protozoan and rickettsial pathogens in ticks collected from infested cattle from Turkey. Pathogens.

[26] Aktas M. (2014). A survey of ixodid tick species and molecular identification of tick-borne pathogens. Vet. Parasitol..

[27] Aktas M., Altay K., Dumanli N., Kalkan A. (2009). Molecular detection and identification of *Ehrlichia* and *Anaplasma* species in ixodid ticks. Parasitol. Res..

[28] Aktas M., Altay K., Ozubek S., Dumanli N. (2012). A survey of ixodid ticks feeding on cattle and prevalence of tick-borne pathogens in the Black Sea Region of Turkey. Vet. Parasitol..

[29] Aktas M., Altay K., Dumanli N. (2006). PCR-based detection of *Theileria ovis* in *Rhipicephalus bursa* adult ticks. Vet. Parasitol..

[30] Aktas M., Ozübek S., Ipek D.N.S. (2013). Molecular investigations of *Hepatozoon* species in dogs and developmental stages of *Rhipicephalus sanguineus*. Parasitol. Res..

[31] Altay K., Aktas M., Dumanli N. (2008). Detection of *Babesia ovis* by PCR in *Rhipicephalus bursa* collected from naturally infested sheep and goats. Res. Vet. Sci..

[32] Aydin M., Aktas M., Dumanli N. (2012). Tick infestations on sheep and goats in the Black Sea Region of Türkiye. Kafkas Univ. Vet. Fak. Derg..

[33] Ozubek S., Aktas M. (2017). Molecular and parasitological survey of ovine piroplasmosis, including the first report of *Theileria annulata* (Apicomplexa: Theileridae) in sheep and goats from Turkey. J. Med. Entomol..

[34] Benedicto B., Ceylan O., Moumouni P.F.A., Lee S.-H., Tumwebaze M.A., Li J., Galon E.M., Liu M., Li Y., Ji S. (2020). Molecular detection and assessment of risk factors for tick-borne diseases in sheep and goats from Turkey. Acta Parasitol..

[35] Ceylan O., Byamukama B., Ceylan C., Galon E.M., Liu M., Masatani T., Xuan X., Sevinc F. (2021). Tick-borne hemoparasites of sheep: A molecular research in Turkey. Pathogens.

[36] Sevinc F., Zhou M., Cao S., Ceylan O., Aydin M.F., Sevinc M., Xuan X. (2018). Haemoparasitic agents associated with ovine babesiosis: A possible negative interaction between *Babesia ovis* and *Theileria ovis*. Vet. Parasitol..

[37] Horak I.G., Heyne H., Williams R., Gallivan G.J., Spickett A.M., Bezuidenhout J.D., Estrada-Peña A. (2018). The Ixodid Ticks (Acari: Ixodidae) of Southern Africa.

[38] Adjou Moumouni P.F., Terkawi M.A., Jirapattharasate C., Cao S., Liu M., Nakao R., Umemiya-Shirafuji R., Yokoyama N., Sugimoto C., Fujisaki K. (2016). Molecular detection of spotted fever group rickettsiae in *Amblyomma variegatum* ticks from Benin. Ticks Tick Borne Dis..

[39] Casati S., Sager H., Gern L., Piffaretti J.-C. (2006). Presence of potentially pathogenic *Babesia* sp. for Human in *Ixodes ricinus* in Switzerland. Ann. Agric. Environ. Med..

[40] McLure A., O’Neill B., Mayfield H., Lau C., McPherson B. (2021). PoolTestR: An R package for estimating prevalence and regression modelling for molecular xenomonitoring and other applications with pooled samples. Environ. Model. Softw..

[41] Sivakumar T., Tuvshintulga B., Zhyldyz A., Kothalawala H., Yapa P.R., Kanagaratnam R., Vimalakumar S.C., Abeysekera T.S., Weerasingha A.S., Yamagishi J. (2018). Genetic analysis of *Babesia* isolates from cattle with clinical babesiosis in Sri Lanka. J. Clin. Microbiol..

[42] Kirvar E., Ilhan T., Katzer F., Wilkie G., Hooshmand-Rad P., Brown D. (1998). Detection of *Theileria lestoquardi* (hirci) in ticks, sheep, and goats using the polymerase chain reaction. Ann. N. Y. Acad. Sci..

[43] Yin H., Liu Z., Guan G., Liu A., Ma M., Ren Q., Luo J. (2008). Detection and differentiation of *Theileria luwenshuni* and *T*. *uilenbergi* infection in small ruminants by PCR. Transbound. Emerg. Dis..

[44] Altay K., Dumanli N., Holman P.J., Aktas M. (2005). Detection of *Theileria ovis* in naturally infected sheep by nested PCR. Vet. Parasitol..

[45] Inci A., Yildirim A., Duzlu O., Doganay M., Aksoy S. (2016). Tick-borne diseases in Turkey: A review based on one health perspective. PLoS Negl. Trop Dis..

[46] Estrada-Peña A., Ayllón N., de la Fuente J. (2012). Impact of climate trends on tick-borne pathogen transmission. Front. Physiol..

[47] Wang Y.Z., Mu L.M., Zhang K., Yang M.H., Zhang L., Du J.Y., Liu Z.Q., Li Y.X., Lu W.H., Chen C.F. (2015). A broad-range survey of ticks from livestock in Northern Xinjiang: Changes in tick distribution and the isolation of *Borrelia burgdorferi sensu stricto*. Parasit. Vectors.

[48] Ceylan O., Uslu A., Ceylan C., Sevinc F. (2021). Predominancy of *Rhipicephalus turanicus* in tick-infested sheep from Turkey: A large-scale survey. Pak. Vet. J..

[49] Uilenberg G. (2006). *Babesia*—A historical overview. Vet. Parasitol..

[50] Bai Q., Liu G., Liu D., Ren J., Li X. (2002). Isolation and preliminary characterization of a large *Babesia* sp. from sheep and goats in the Eastern part of Gansu Province, China. Parasitol. Res..

[51] Sevinc F., Sevinc M., Ekici O.D., Yildiz R., Isik N., Aydogdu U. (2013). *Babesia ovis* infections: Detailed clinical and laboratory observations in the pre-and post-treatment periods of 97 field cases. Vet. Parasitol..

[52] Ceylan O., Sevinc F. (2020). Endemic instability of ovine babesiosis in Turkey: A country-wide sero-epidemiological study. Vet. Parasitol..

[53] Altay K., Dumanli N., Aktas M. (2012). A study on ovine tick-borne hemoprotozoan parasites (*Theileria* and *Babesia*) in the East Black Sea Region of Turkey. Parasitol. Res..

[54] Zhou M., Cao S., Sevinc F., Sevinc M., Ceylan O., Ekici S., Jirapattharasate C., Moumouni P.F.A., Liu M., Wang G. (2017). Molecular detection and genetic characterization of *Babesia*, *Theileria* and *Anaplasma* amongst apparently healthy sheep and goats in the Central Region of Turkey. Ticks Tick Borne Dis..

[55] Aydin M.F., Aktas M., Dumanli N. (2015). Molecular identification of *Theileria* and *Babesia* in ticks collected from sheep and goats in the Black Sea Region of Turkey. Parasitol. Res..

[56] Friedhoff K. (1997). Tick-borne diseases of sheep and goats caused by *Babesia*, *Theileria* or *Anaplasma* spp. Parasitologia.

[57] Ozubek S., Aktas M. (2018). Molecular evidence for a novel species of *Babesia* in unfed *Rhipicephalus sanguineus sensu lato* (Acari: Ixodidae). J. Med. Entomol..

[58] Hamid P.H., Cahyadi M., Wardhana A.H., Sawitri D.H., Setya N.N.R., Insyariati T., Kurnianto H., Hermosilla C.R. (2022). First autochthonous report on cattle *Babesia naoakii* in Central Java, Indonesia, and identification of *Haemaphysalis bispinosa* ticks in the investigated area. Pathogens.

[59] Futse J.E., Ueti M.W., Knowles D.P., Palmer G.H. (2003). Transmission of *Anaplasma marginale* by *Boophilus microplus*: Retention of vector competence in the absence of vector-pathogen interaction. J. Clin. Microbiol..

[60] Ringo A.E., Aboge G.O., Adjou Moumouni P.F., Hun Lee S., Jirapattharasate C., Liu M., Gao Y., Guo H., Zheng W., Efstratiou A. (2019). Molecular detection and genetic characterisation of pathogenic *Theileria*, *Anaplasma* and *Ehrlichia* species among apparently healthy sheep in Central and Western Kenya. Onderstepoort J. Vet. Res..

[61] Aktaş M., Altay K., Dumanli N. (2005). Survey of *Theileria* parasites of sheep in eastern Turkey using polymerase chain reaction. Small Rumin. Res..

[62] Ahmed J., Yin H., Bakheit M., Liu Z., Mehlhorn H., Seitzer U. (2011). Small ruminant theileriosis. Prog. Parasitol..

[63] Büscher G., Friedhoff K.T., El-Allawy T.A.A. (1988). Quantitative Description of the development of *Babesia ovis* in *Rhipicephalus bursa* (hemolymph, ovary, eggs). Parasitol. Res..

[64] Neitz W.O. (1972). The experimental transmission of *Theileria ovis* by *Rhipicephalus evertsi mimeticus* and *R*. *bursa*. Onderstepoort J. Vet. Res..

